# Anatomic Feasibility of In-Situ Fenestration for Isolate Left Subclavian Artery Preservation during Thoracic Endovascular Aortic Repair Using an Adjustable Needle Puncturing System

**DOI:** 10.3390/jcm13010162

**Published:** 2023-12-27

**Authors:** Gabriele Piffaretti, Marco Franchin, Aroa Gnesutta, Tonia Gatta, Filippo Piacentino, Nicola Rivolta, Chiara Lomazzi, Daniele Bissacco, Federico Fontana, Santi Trimarchi

**Affiliations:** 1 Vascular Surgery—Department of Medicine and Surgery, University of Insubria School of Medicine, 21100 Varese, Italy; marco.franchin@hotmail.it (M.F.); nicola.rivolta@asst-settelaghi.com (N.R.); 2 ASST Settelaghi University Teaching Hospital, 21100 Varese, Italy; 3 Interventional Radiology—Department of Medicine and Surgery, University of Insubria School of Medicine, ASST Settelaghi University Teaching Hospital, 21100 Varese, Italy; 4 Vascular Surgery, Cardio Thoracic Vascular Department, Foundation IRCCS Ca’ Granda Ospedale Maggiore Policlinico, 20122 Milan, Italysanti.trimarchi@unimi.it (S.T.); 5 Department of Clinical Sciences and Community Health, University of Milan, 20122 Milan, Italy

**Keywords:** in-situ fenestration, left subclavian artery fenestration, adjustable needle, “zone 2” TEVAR

## Abstract

Objectives: To evaluate the feasibility of thoracic endovascular aortic repair (TEVAR) using the Ankura™ device (Lifetech Scientific, Shenzhen, China) with left subclavian artery (LSA) in-situ fenestration (ISF) using an adjustable puncture device system. Methods: It is a single center, retrospective, financially unsupported cohort study of TEVAR performed from 16 February 2007 to 10 January 2023. Inclusion criteria were isolate LSA revascularization for elective or urgent/emergent “zone 2” TEVAR, and the availability of the preoperative computed tomography angiography. Results: Post-hoc analysis identified 52 TEVARs. There were 39 (75.0%) males, and 13 (25.0%) females: median age was 74.5 years (IQR, 65.5–78). Index TEVAR was performed for atherosclerotic aneurysm in 27 (51.9%) cases, dissection-related diseases in 18 (34.6%), penetrating aortic ulcer in 5 (9.6%), and blunt traumatic aortic injury in 2 (3.8%). Access-vessel feasibility rate of TEVAR using the Ankura™ device would have been 98.1% (51/52). Considering the morphology of the aortic arch, ISF TEVAR feasibility would have been 61.5% (32/52). Binary logistic regression analysis identified LSA angulation (OR: 1.1, 95%CI: 1.03–1.14, *p* = 0.003) to be associated with ISF feasibility using this endograft and a self-centering adjustable needle-based puncture device. Conclusions: Potential feasibility of TEVAR using the Ankura™ endograft with ISF using a self-centering adjustable needle system was 61.5%. Left subclavian artery angulation seems to be the most important and limiting anatomical constraint.

## 1. Introduction

Left subclavian artery (LSA) management during open repair of distal aortic arch disease has generated unique anatomic and technical concerns that made it a procedure with high neurologic complications and operative mortality [1]. Left subclavian artery management generated a lot of debate also at the beginning of the thoracic endovascular aortic repair (TEVAR) era [2]. Recent meta-analyses demonstrated that revascularization of the LSA is associated with decreased risks of cerebrovascular accidents, spinal cord ischemia (SCI), and left upper limb ischemia but at the cost of worrying local complications [3,4,5]. Different alternative endovascular solutions, such as the parallel grafts techniques, customized fenestrated or scalloped or branched endograft, have been designed to challenge left carotid-LSA bypass or transposition [6,7,8,9,10,11,12]. Among total endovascular solutions, the former are versatile but have inconsistent durability, while the latter offer encouraging results but are complex procedures and require long manufacturing time. In-situ fenestration (ISF) of thoracic endograft, which is not a new concept, combines several attractive aspects: it is an anatomical anterograde reconstruction and thus mimics open repair, it is an intuitive technique because it is based on simple technical steps, and it is versatile for urgent/emergent scenarios [13,14]. Among different ISF techniques, a self-centering adjustable needle-based puncture device was recently designed to improve the safety and success rate of ISF [15,16]. The aim of the present study was to estimate the theoretical anatomic feasibility of LSA ISF TEVAR using this device in a real-life cohort of thoracic aortic diseases, by retrospectively analyzing a cohort of “zone 2” TEVAR already operated with different techniques to preserve the LSA. 

## 2. Methods

### 2.1. Study Cohort

**Figure 1:**
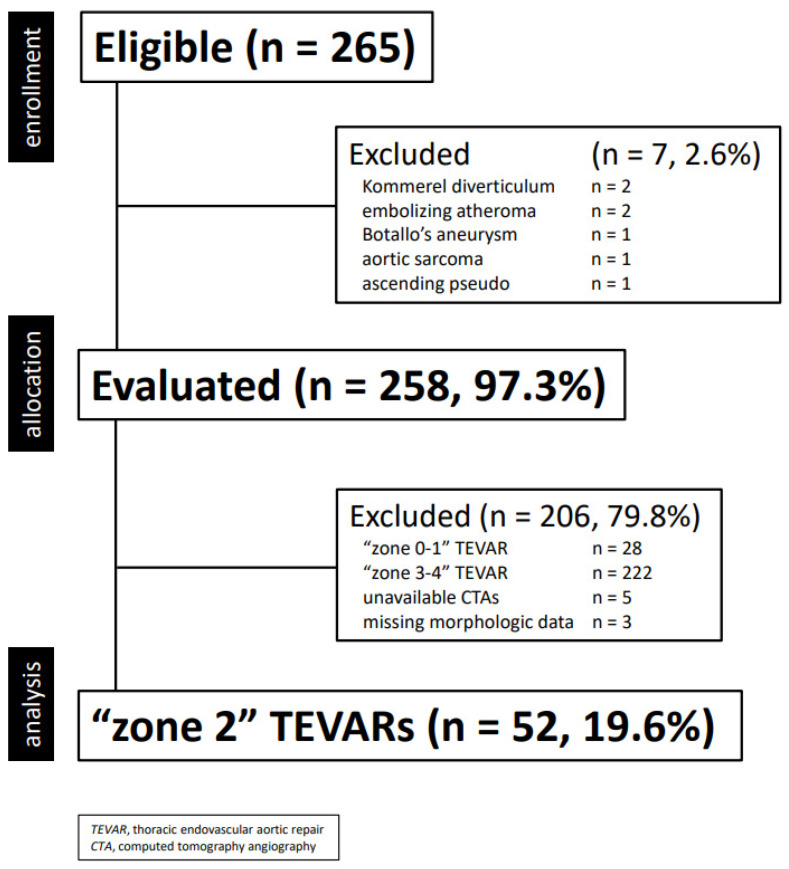
Consort diagram of all thoracic aortic diseases treated with thoracic endovascular aortic repair (2001–2022, *n* = 255).

This is a single center, retrospective, financially unsupported physician-initiated cohort study [17]. Clinical data were collected in a dedicated, anonymized institutional database and analyzed retrospectively. Information collected included demographics, co-morbidities, morphologic characteristics of the aortic lesion, type of intervention and endograft, as well as postoperative events (complications, death, reintervention) during hospitalization and follow-up. For this study, only TEVAR performed between 16 February 2007 and 10 January 2023 were extracted and included in the final cohort according to the following criteria (Figure 1):

- elective or urgent/emergent TEVAR
- isolate LSA revascularization during “zone 2” TEVAR
- available preoperative computed tomography angiography (CTA) stored in a retrievable electronic archive performed within six months TEVAR, and including scans of the supra-aortic-trunks and Willis

Circle

- All examinations carried out in the same center.

Exclusion criteria were the following ones:

- LSA preservation/revascularization for “zone 0–1” TEVAR
- supra-aortic anatomic variants
- missing preoperative CTA
- missing clinical and/or procedural data.

### 2.2. Device Description and IFUs

**Figure 2:**
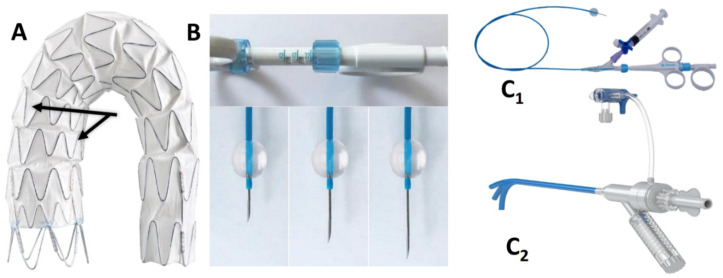
The two different waves (*black arrows*) of the nitinol frame of then Ankura^TM^ endograft (**A**). FuThrough^TM^ needle system (**B**,**C_1_**) adjustable in length. Fustar^TM^ (**C_2_**) steerable sheath.

**Figure 3:**
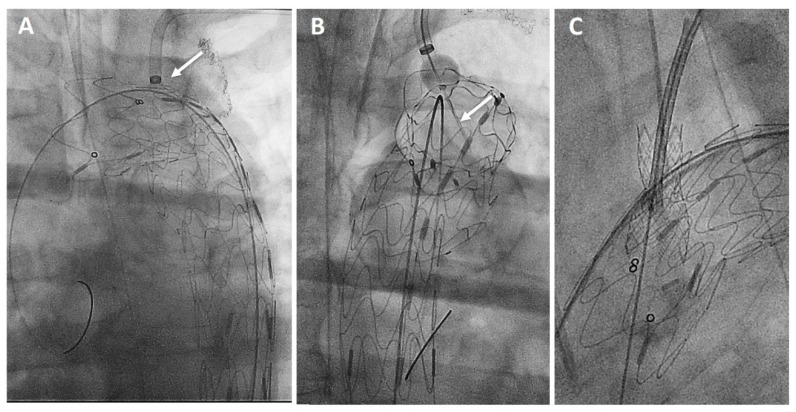
Procedural steps: perpendicular adjustment (*white arrow*) of the puncturing system (**A**). Needle penetration (*white arrow*) at the outer curvature of the aortic endograft (**B**). Bridging stent-graft protruding antegrade toward the ascending aorta (**C**).

The Ankura^TM^ (Lifetech Scientific; Shenzhen, China) endograft is made of a self-expanding nitinol stent associated with an ultra-thin double membrane (inner and outer) of expanded polytetrafluoroethylene (ePTFE) through a heat-sealing process without sutures on the main body. A longitudinal supporting strut on the greater curvature provides axial support. The proximal edge has a free flow with six apices covered with ePTFE; the supporting nitinol frame has a specific configuration with smaller wave at the inner part of the endograft which progressively enlarged toward the outer curve to better adapt flexibility to the aortic arch curvature (Figure 2A). The hydrophilic coated delivery system ranges from 20F (outer diameter), for endograft of 20–36 mm in diameter, to 22F (outer diameter) for diameter of 38–46 mm. According to the instruction for use (IFU), it requires ≥15 mm of healthy proximal aortic neck, and an aortic inner diameter in the range of 16–44 mm. The puncture system (FuThrough^TM^—Lifetech Scientific; Shenzhen, China) consists of a self-centering balloon catheter equipped with a 20 G needle that is adjustable in length (Figure 2B). Our institution technique has consolidated steps: after endograft deployment, a steerable (Fustar^TM^—Lifetech Scientific; Shenzhen, China) sheath is inserted through a left axillary artery surgical cutdown to be positioned as perpendicular as possible to the greater curvature of the aortic arch, possibly in direct contact with the outer curvature of the endograft (Figure 3A). The balloon at the tip of the adjustable puncture needle is inflated to stabilize the system. The penetration depth was preselected on this device. After pushing the trigger of the puncture needle, the fenestration is created. An 0.018 guidewire is then advanced into the ascending (or descending), and the fenestration gently, progressively dilated using 4 mm-up to 8 mm diameter non-compliant balloons (Figure 3B). Finally, ISF is connected with a balloon-expandable chrome cobalt ePTFE stent-graft (BeGraft/BeGraft^®^ plus—Bentley; Hechingen, Germany) with approximately 10% oversize, possibly deployed antegrade up to 5 mm inside the fenestration (Figure 3C).

### 2.3. Imaging Analysis

**Figure 4:**
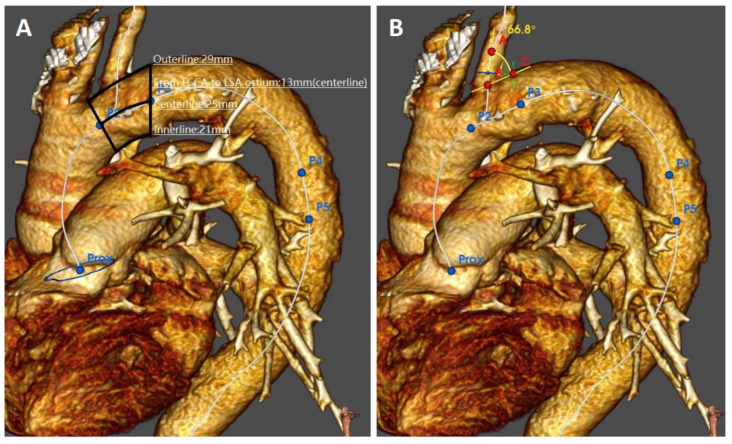
Preoperative imaging evaluation of the aortic arch morphologic features: proximal landing zone lengths (**A**) and angulation of the left subclavian artery take-off (**B**).

All computed tomography angiographies (CTAs) were carried out with a spectral CT (IQon^®^ Spectral CT—Philips; Amsterdam, The Netherlands). Datasets of all CTAs were transferred to a dedicated workstation equipped with software for vascular reconstructions and volume calculation (IntelliSpace^®^ Portal 9.0—Philips, Amsterdam; The Netherlands). Aortic disease features were evaluated retrospectively on axial, multiplanar and volume rendering 3D images by a panel of two vascular interventional radiologists (FF and FP) in association with a vascular surgeon (GP), all having ≥20 years of experience with both CTA imaging and associated TEVAR planning. The feasibility of TEVAR was adjudicated by consensus of three evaluators blind to the patients’ identity. Access feasibility was determined by measuring both the inner and the outer diameter of the largest iliac-femoral axis. The narrowest point on the larger iliac-femoral axis was chosen as the minimum access diameter. Aortic feasibility was determined by the aortic neck diameters, lengths, and angles at the level of the supra-aortic trunks. Diameters were measured outer to the outer layer at the intended proximal landing “zone 2”. Lengths were calculated as inner/centerline/outer curvatures stretched length between the distal edge of left common carotid artery and the proximal edge of the LSA or aortic neck (Figure 4A,B). The distance between the LSA and the ipsilateral vertebral artery take-off was intended as stretched center-line length between the take-off of LSA and the origin of the vertebral artery. Left subclavian artery vessel feasibility was determined by the diameter, length to the level of the ipsilateral vertebral artery, and angulation form the aortic arch. Angulations were evaluated in the best multiplanar projection of the arch.

### 2.4. Definitions and Primary Outcomes

Comorbidities and risk factors were classified according to the Society for Vascular Surgery (SVS) ad hoc committee on TEVAR reporting standards [18]. Morphologic characteristics and outcomes were defined according to EACTS best practice guidelines for reporting treatment results in the thoracic aorta [19,20]. Specifically for this study, the primary outcome was the theoretical anatomic feasibility of LSA ISF TEVAR using a new self-centering adjustable needle-based puncture system.

### 2.5. Outcomes and Statistical Analysis [21]

Statistical analysis was performed with SPSS, release 27.0 for Windows (IBM SPSS Inc., Chicago, IL, USA). Continuous variables were tested for normal distribution by the Shapiro-Wilk test. Continuous variables that presented normally distributed were reported as mean ± standard deviation (SD) with range, otherwise with medians and interquartile (IQR) range (25th–75th percentile interval) were applied. Categorical variables were reported as counts and percentages, and analysed with the Pearson’s χ^2^ test or Fisher’s exact test whether the expected cell frequencies were <5. Independent samples Student’s *T*-test was used for continuous variables. Interobserver agreement was calculated for measurements by using a mixed model of intraclass correlations for absolute agreement. Values were expressed by adjusted squared correlation coefficients (*r*^2^) to circumstantiate the degree of dependence even more precisely. The strength of the association of variables with non-feasibility was estimated by calculating the odd ratio (OR) and 95% confidence intervals [(95%CI): significance criteria 0.20 for entry, 0.05 for removal)]. Model discrimination was evaluated by using the area under the receiver operating characteristic (AUROC) curve. All reported *p* values were two-sided; *p* value < 0.05 was considered significant.

## 3. Results

### 3.1. Study Cohort

The final cohort included 52 (19.6%) patients. There were 39 (75.0%) males, and 13 (25.0%) females: median age was 74.5 years (IQR, 65.5–78). Index TEVAR was performed for atherosclerotic aneurysm in 27 (51.9%) cases, type B aortic dissection in 18 (34.6%), penetrating aortic ulcer in 5 (9.6%), and blunt traumatic injury in 2 (3.8%). Elective intervention was performed in 35 (67.3%) cases; emergent TEVAR was performed in 17 (32.7%) cases because of rupture (*n* = 8), or malperfusion (*n* = 6). During index TEVAR, LSA was revascularized with carotid-subclavian bypass in 34 (65.4%) cases, with a parallel graft technique in 8 (15.4%), fenestration in 5 (9.6%, in situ *n* = 4), and single-branched in 1 (1.9%); it was simply covered in 4 (7.7%). Additional supra-aortic trunks procedure included transposition of an aberrant left vertebral artery in 4 cases (7.7%), and bailout left common carotid artery stenting in 1 (1.9%) case.

### 3.2. Results of Index TEVAR

Primary technical success of index TEVAR was obtained in 51 (98.1%) cases: one patient treated in emergency for rupture died intraoperatively due to consequences of hemorrhagic shock despite a successful deployment and sealing. Early (≤30 days) mortality occurred in 4 (7.7%) cases. The median of follow-up was 59 months (IQR, 9–96.25). Estimated survival was 95% (SE: 0.03, 95%CI: 83.7–98.7) at 12 and 60 months. Estimated freedom from aorta-related reintervention was 95% (SE: 0.03, 95%CI: 83.7–98.7) at 12 months, 90% (SE: 0.03, 95%CI: 76.3–90) at 36 months, and 82% (SE: 0.07, 95%CI: 65.7–91.9) at 60 months. The LSA management did not affect survival (Log-rank = 0.837) or aorta-related reintervention during the follow-up (Log-rank = 0.231). A total of 8 (16.7%) aorta-related reinterventions were performed: none was LSA-related but performed distal to the index TEVAR in all but one case (acute type A aortic dissection).

### 3.3. In-Situ Fenestration TEVAR Feasibility Evaluation

**Figure 5:**
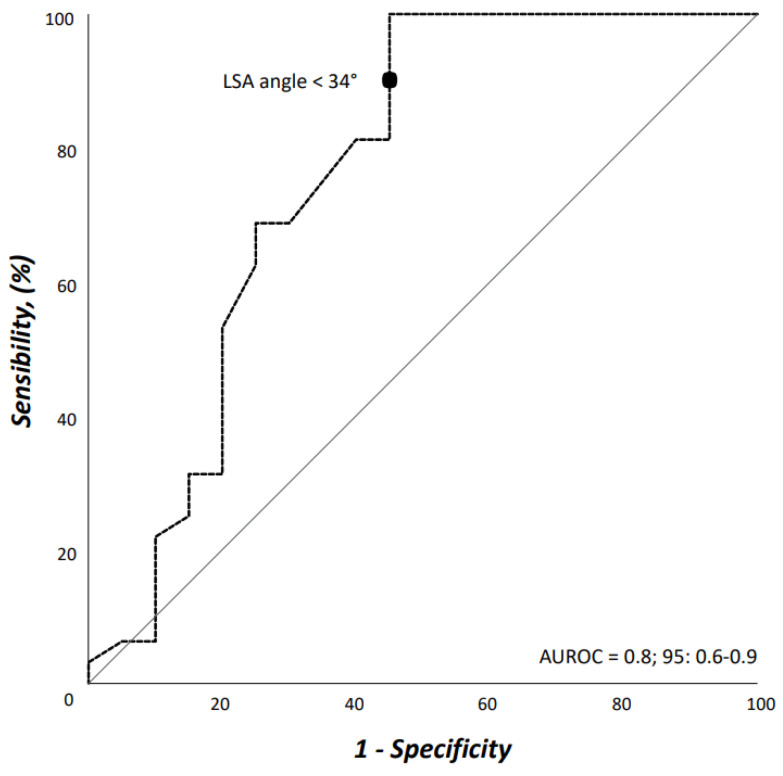
Area under the receiver operating characteristic (AUROC) curve for the multivariable model including left subclavian artery angulation from the aortic arch.

**Figure 6:**
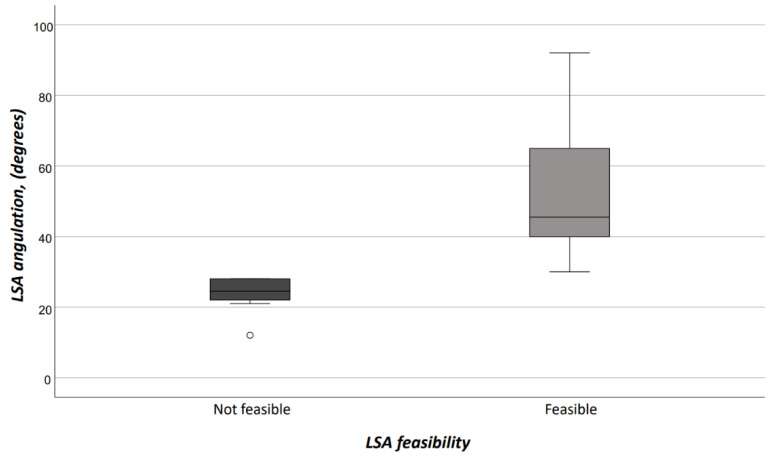
In-situ fenestration according to LSA feasibility. (LSA = left subclavian artery).

Interobserver agreement on both access vessels and aortic arch morphologic features was high (*r* = 0.98). Demographics, comorbidities, and risk factors are reported in Table 1. There were no differences between patients who would have received TEVAR with ISF using the Ankura™ device, and those who would have been considered unfit for this type of TEVAR; in particular, there was no difference in patients potentially treated in urgent/emergent scenarios [*n* = 12 (37.5%) vs. 5 (25.0%), OR: 1.8, *p* = 0.383]. The differences of morphologic features in the two groups are summarized in Table 2. Considering the size of the device that should have been implanted at the index procedure, the overall access-vessel correlated feasibility rate of TEVAR using the Ankura™ device would have been 98.1% (51/52). The patient with no morphological requirement for TEVAR with this device would have required an aorto-bifemoral bypass graft to overcome this anatomical issue as indeed originally occurred during index TEVAR. The mean aortic endograft size was 32 mm ± 4 (range, 25–42) during the index TEVAR, and 36 mm ± 4 (range, 28–46) if TEVAR with the ISF using the Ankura™ device would have been used. No case would have been rejected for ISF TEVAR. Out of the 20 (38.5%) cases considered unfit for ISF, there were 11 (55.0%) cases of type III aortic arch, 5 (25.0%) type II aortic arch, and 4 (20.0%) type I aortic arch (*p* = 0.359). The presence of an aberrant left vertebral artery in 4 (7.7%) patients would have not ruled out the possibility of performing a TEVAR with the ISF that, however, would have required the transposition of the left vertebral artery as indeed happened in the index TEVAR. Considering all morphologic features, ISF TEVAR using the Ankura™ device would have been 61.5% (32/52). Binary logistic regression analysis showed that LSA angulation (OR: 1.1, 95%CI: 1.03–1.14, *p* = 0.003) negatively impacted ISF with this technique. The AUROC analysis showed a fairly good discrimination for this multivariable model (Figure 5). In particular, an LSA angle of 34° yielded a 91% sensitivity and 45% specificity, equaling an 78.6% positive predictive value with an accuracy of 76.9% (Figure 6).

## 4. Discussion

Our main findings are twofold: first, LSA ISF TEVAR using a self-centering adjustable needle-based puncture device in combination with the Ankura™ endograft has a theoretically feasibility in nearly 2/3 of the cases previously treated with conventional carotid-subclavian bypass and TEVAR, or TEVAR with the parallel graft technique. Secondly, LSA angulation is the most limiting anatomic constraint to perform this ISF technique.

Three recent meta-analyses of TEVAR with and without revascularization of the LSA coverage showed that revascularization was protective against neurologic complications or mortality [3,4,5]. For these reasons, two cardiovascular societies recently endorse LSA revascularization during “zone 2” TEVAR [20,22]. Nonetheless, modern clinical surgery is committed toward the development of minimally invasive techniques while maintaining effectiveness and durability. While surgical bypass has gained a reputation of effective and durable procedure, it has been associated with worrying local and central neurological complications [3]. The initial enthusiasm for the parallel graft techniques has given way to widespread skepticism because of the high rate of TEVAR-related complications and reoperations already in the medium term [6,23]. Branched and fenestrated endografts bring the best of the minimally invasive nature of endovascular solution with the possibility to be tailored for each patient’s anatomy; however, they are expensive and technically demanding with no immaculate results in terms of neurologic sequalae and reintervention either [24,25]. Despite it being performed for the very first time two decades ago, recently ISF has witnessed rejuvenated interest due to the simpleness and versatility especially in urgent/emergent settings [13,14,15,26]. In our analysis, no demographic parameters would have precluded performing ISF; moreover, it would have been equally feasible in urgent/emergent scenarios. Lastly, despite potential differences which may exist across the country, at least in our country ISF is currently cheaper than branched and fenestrated endografts.

Two systematic reviews ascertained the high technical success rates and the satisfactory results at short-term with different ISF techniques [16,27,28]. Our experience is the first to evaluate the anatomic feasibility of ISF TEVAR with a self-centering adjustable needle. Most of the potential failures described with endovascular technologies can be ascribed to the inadequacy of the access vessels, and the configuration of the aortic arch [26]. The 61.5% feasibility rate estimated in our cohort compares well with the 43%–69% reported in other feasibility-type studies [29,30]. Of note, in our analysis, access vessels viability has not nearly precluded the feasibility of the ISF with the Ankura™ endograft: overall, at least one of the two access vessel side was viable for this device. Sure, we have evaluated a technique that requires a standard off-the-shelf device; however, using a device that does not incorporate side branches reduces the issues determined by a bulky device [30]. Indeed, in our experience the most important anatomic constraint for using ISF was the aortic arch configuration, a finding that is a common issue also for other types of complex TEVAR technologies to be used in this area [26]. Among the different anatomic features, LSA diameter and the distance of the ipsilateral vertebral artery were not of concern; instead, acute angulation of the LSA significantly hampered this type of ISF. Our data find support in the experience of Shu et al. [26] who reported that both acute take-off angle of the LSA and the severe tortuosity rendered it difficult for the needle to pierce the fabric. This observation emphasizes the fact that a type III aortic arch is not a contraindication per se to perform ISF. Both the easier structural endograft configuration and the conformability of the ISF may play an important role in determining technical feasibility of this technique. These aspects make ISF, and this specific technique, an attractive alternative during “zone 2” TEVAR. A technical variant of ISF proposed in the Literature has been laser mediated ISF, with different experiences reporting satisfactory results either in terms of technical success or clinical results [14,31]. Nevertheless, an interesting experimental study demonstrated how laser ISF seems to expose fabric to a higher risk of mechanical resistance loss and leakage at the stented fenestration interface [32]. This is the main reason why we believe that mechanical ISF using this specific methodology, a position that may be supported by the analysis of the quality of mechanical needle assisted ISF: Li et al. [33] clearly demonstrated that the use of this latter technique in association with gradual balloon dilation when using the Ankura™ endograft are crucial for fashioning high-quality fenestrations. Indeed, continuous improvement of the puncture device as well as further anatomic analyses and calculations, such as the creation of a dedicated LSA tortuosity index, will help to identify the best candidates for this ISF technique.

Considering the potential perspective applicability of this procedure with these new devices, one of the important (e.g., potential) drawbacks lies in the absence of reinforced fenestration like occurs in custom-made devices, or already incorporated in single-side branch devices that reasonably and intuitively may be better perceived in terms of EG integrity [7,9,34]. However, not only does the ISF technique perfectly mimic the principle of open surgery at “zone 2”, there are promising data showing the effectiveness and durability of this procedure [26,35] giving it dignity to be implemented in the daily practice armamentarium of cardiovascular operators. Moreover, there are bench tests that also documented the stability of the EG-bridging stent-graft interaction with no problem in terms of dislodgment, especially when coupled with specific bridging stent-graft [36]. Last but not least, it is an off-the-shelf solution with, therefore, no waste of time for EG manufacturing and cheaper financial burden.

### Limitations

This study has several limitations. First, it is retrospective in nature, and it is a theoretical evaluation of the feasibility of this technique. Institutional databases rely solely on accurate site reporting; thus, it is possible that investigators might have not identified all covariates correlated to the aortic anatomy or endovascular procedures. However, missing data were not defaulted to negative, and denominators reflect only reported cases: for these 52 patients, 3328 overall data were collected through 64 variables, with an overall missing data rate of 0.8%. Second, it has sampling bias: alternative fenestration techniques were not investigated for comparison, and the small number of patients included in this cohort may introduce a serious bias in the ascertainment of findings. Third, while we attempted to correct for potential confounders using multivariate analyses, the small number of patients and events makes results of the multivariate model not generalizable: the absence of differences could reflect a type II error. Finally, the anatomical feasibility is calculated based on the current IFUs of the Ankura™ endograft, which may change in the future. All these limitations may not allow for generalizability of our findings; notwithstanding, our data compares well with the available literature because of the lack of data correlated with feasibility of TEVAR with ISF.

## 5. Conclusions

In our experience, ISF TEVAR using the Ankura™ device in addition with the self-centering adjustable needle system had a potential applicability of 61.5% within an historical cohort treated with conventional carotid-subclavian bypass or parallel graft technique. Among anatomic constraints, LSA angulation seems to be the strongest morphological feature limiting the application of this specific technique.

## Figures and Tables

**Table 1:** Demographic data, coexisting comorbidities, and risk factors of the entire cohort.

	ISF—Yes	ISF—No	*p*
	(*n* = 32)	(*n* = 20)	
*Demographics*			
Gender, M:F (ratio)	26:6	13:7	0.208
Age, mean ± SD (range)	70 ± 10 (45–81)	72 ± 9 (52–86)	0.187
Age > 80 y	4 (12.5)	4 (20.0)	0.695
*Comorbidities*			
Hypertension	31 (96.9)	20 (100)	1.0
CAD	3 (9.4)	2 (10.0)	1.0
COPD ^a^	12 (37.5)	6 (30.0)	0.766
CKD ^b^	2 (6.2)	2 (10.0)	0.634
*Risk factors*			
Urgent/emergent/emergent, *n* (%)	12 (37.5)	5 (25.0)	0.383
Proximal aortic surgery	8 (25.0)	6 (30.0)	0.758
Thoraco-abdominal	10 (31.2)	7 (35.0)	1.0
SVS score, median (IQR) ^c^	5 (4–8)	5 (4–8)	0.848
*Aortic pathology*, *n* (*%*)			0.389
Aneurysm	18 (56.3)	14 (70.0)	
Dissection/Trauma	14 (43.7)	6 (30.0)	

ISF, in-situ fenestration; *n*, number; M, male; F, female; SD, standard deviation; IQR, interquartile range; *SVS*, Society for Vascular Surgery; ^a^ http://www.goldcopd.org (accessed on 23 October 2023); ^b^ Ann Intern Med 2009; 150:604–612; ^c^ J Vasc Surg 2017; 66:1321–1333.

**Table 2:** Morphologic features of the aortic lesions in the entire cohort.

	ISF—Yes	ISF—No	*p*
	(*n* = 32)	(*n* = 20)	
*Morphologic features*, *median (IQR)*			
right femoral, mean ± SD (mm)	9 ± 2 (7–13)	7.5 ± 2 (5–11)	0.004
left femoral, mean ± SD (mm)	8.5 ± 2 (6–12)	8 ± 2 (4–12)	0.083
right external iliac, mean ± SD (mm)	9 ± 1.5 (7–12)	8 ± 2 (5–11)	0.003
left external iliac, mean ± SD (mm)	9 ± 2 (4–12)	8 ± 2 (4–11)	0.011
right common iliac, mean ± SD (mm)	11 ± 3 (4–20)	11.5 ± 3 (6–15)	0.894
left common iliac, mean ± SD (mm)	11.5 ± 2.5 (7–15)	11 ± 2.5 (6–15)	0.492
aortic arch, *n* (%)			0.359
type I	6 (18.8)	4 (20.0)	
type II	14 (43.7)	5 (25.0)	
type III	12 (37.5)	11 (55.0)	
LSA angulation, mean ± SD (degrees)	51 ± 15 (32–92)	35 ± 17 (12–83)	0.009
PLZ outer curve, mean ± SD (mm)	26 ± 10 (11–50)	29 ± 14 (9–59)	0.580
PLZ centerline, mean ± SD (mm)	23 ± 8 (10–42)	23 ± 10 (8–43)	0.902
PLZ inner curve, mean ± SD (mm)	20 ± 7 (7–39)	19 ± 7 (5–33)	0.499
aortic prox neck, mean ± SD (mm)	31 ± 3 (25–37)	34 ± 4 (27–42)	0.004
endograft diameter, mean ± SD (mm)	35 ± 3 (28–42)	38 ± 4 (32–46)	0.005

ISF, in-situ fenestration; *n*, number; IQR, interquartile range; SD, standard deviation; LSA, Left subclavian artery; PLZ, proximal landing zone.

## Data Availability

The data underlying this article will be shared on reasonable request to the corresponding author.
